# Oxidative Stress in ESRD Patients on Dialysis and the Risk of Cardiovascular Diseases

**DOI:** 10.3390/antiox9111079

**Published:** 2020-11-03

**Authors:** Jacek Rysz, Beata Franczyk, Janusz Ławiński, Anna Gluba-Brzózka

**Affiliations:** 1 Department of Nephrology, Hypertension and Family Medicine, Medical University of Lodz, 90-419 Łódź, Poland; jacek.rysz@umed.lodz.pl (J.R.); bfranczyk-skora@wp.pl (B.F.); 2 Department of Urology, Institute of Medical Sciences, Medical College of Rzeszow University, 35-959 Rzeszow, Poland; janlaw@wp.pl

**Keywords:** end-stage renal disease, oxidative stress, cardiovascular disease, antioxidant therapy

## Abstract

Chronic kidney disease is highly prevalent worldwide. The decline of renal function is associated with inadequate removal of a variety of uremic toxins that exert detrimental effects on cells functioning, thus affecting the cardiovascular system. The occurrence of cardiovascular aberrations in CKD is related to the impact of traditional risk factors and non-traditional CKD-associated risk factors, including anemia; inflammation; oxidative stress; the presence of some uremic toxins; and factors related to the type, frequency of dialysis and the composition of dialysis fluid. Cardiovascular diseases are the most frequent cause for the deaths of patients with all stages of renal failure. The kidney is one of the vital sources of antioxidant enzymes, therefore, the impairment of this organ is associated with decreased levels of these enzymes as well as increased levels of pro-oxidants. Uremic toxins have been shown to play a vital role in the onset of oxidative stress. Hemodialysis itself also enhances oxidative stress. Elevated oxidative stress has been demonstrated to be strictly related to kidney and cardiac damage as it aggravates kidney dysfunction and induces cardiac hypertrophy. Antioxidant therapies may prove to be beneficial since they can decrease oxidative stress, reduce uremic cardiovascular toxicity and improve survival.

## 1. Introduction

Chronic kidney disease (CKD) is highly prevalent worldwide (between 11 and 13%), and it is most frequent in developed countries in Europe, USA, Canada, and Australia [1,2]. CKD progresses as GFR decreases and this process results from the deterioration of kidney function, which greatly influences body homeostasis and leads to biological and clinical dysfunctions, including the disturbances in cellular energetic metabolism, protein malnutrition, change in nitrogen input/output, insulin resistance, and significant increase in the synthesis of inflammation/oxidative stress mediators [2]. Finally, it progresses to end-stage renal disease (ESRD) and ends up with the necessity for renal replacement therapy (hemodialysis or peritoneal dialysis) or renal transplantation [3]. The decline of renal function is associated with inadequate removal of a variety of uremic toxins that should be excreted by the kidney. Due to the fact these substances are biologically active, they are both the cause and consequence of CKD [4]. According to studies, uremic toxins exert a detrimental effect on cells involved in the functioning of myocardium and vessels, including smooth muscle cells, endothelial cells (ECs), and platelets leucocytes, thus affecting the cardiovascular system [5]. Cardiovascular diseases (CAD) are the most frequent cause of death for patients with all stages of renal failure, and they are present in >50% of patients undergoing dialysis [4]. 

## 2. Cardiovascular Diseases in ESRD Patients 

The occurrence of cardiovascular aberrations in CKD is related to the influence of traditional risk factors (hypertension, diabetes mellitus, etc.) and also non-traditional CKD-associated risk factors, including anemia; inflammation; mineral and bone disease abnormalities; oxidative stress; the presence of some uremic toxins; and factors related to the type, frequency of dialysis and the composition of fluid used during the procedure [6,7,8,9]. Increased CAD risk in ESRD patients is also related to the fact that this disease frequently results from hypertension and diabetes mellitus [4]. The prevalence of coronary heart disease and ventricular hypertrophy has been reported to be 40% and 70%, respectively, in renal replacement therapy patients [10]. The likelihood of developing CAD increases linearly in patients whose glomerular filtration rate (eGFR) decreases below ~60–75 mL/min/1.73 m^2^, and CAD mortality risk in patients with CKD stages G3a to G4 is twice or three times higher compared to patients without CKD [6,11,12]. Also, the prevalence of clinical manifestations of CAD as well as the frequency of arteriosclerosis, LVH, large-vessel coronary disease, myocardial fibrosis, and microvascular disease rises along with the decline of eGFR. The mortality of patients with end-stage renal failure is considerably higher (20 times higher in HD patients) than in the general population [13,14]. Nearly 50% of patients undergoing hemodialysis die of cardiovascular causes [15]. The rate of cardiovascular death is increasing with aggravating kidney impairment even after the adjustment for common CAD risk factors [6,11,16]. Hemodialysis offers a temporary solution for renal dysfunction since it replaces some filtration functions of the kidney, however, it does not diminish morbidity and mortality related to inflammation and its complications, such as cardiovascular disease or oxidative stress [17]. The long-term survival and prognosis of patients undergoing dialysis after acute myocardial events are poor [18]. Numerous studies confirmed that the calcification of the sub-intima and media of large vessels increased the risk of all-cause and cardiovascular mortality in this group [19]. The autopsy studies of patients with chronic kidney disease have revealed the presence of more advanced atherosclerotic plaques, more aggravated medial calcifications of these lesions compared to patients without kidney impairment, as well as inflammation in the coronary plaques [20,21,22,23]. High mortality of dialysis patients may also be ascribed to sudden deaths, which are associated with arrhythmia triggered by shifts in volume, electrolytes and drug concentrations in patients with a myocardial disease (LVH and heart failure) [6]. Mortality related to sudden death and heart failure decreases after kidney transplantation [6]. The impairment of renal clearance in CKD results in the accumulation of toxins, such as p-cresol and indoxyl sulphate, which not only stimulate the expression of intercellular adhesion molecule-1 (ICAM) and monocyte chemotactic protein-1 (MCP-1), but also induce the activation of NADPH oxidase, increasing the production of reactive oxygen species, as well as pose surplus cardiovascular risk in CKD [24,25]. Also, uremic immune dysfunction is considerably associated with high rate of premature mortality in ESRD patients due to the impact on cardiovascular and infectious complications [26]. Malnutrition–inflammation–atherosclerosis (MIA) syndrome is an important complication observed in patients with advanced stages of CKD, and it is accompanied by higher incidence of CVD and rapid progression of atherosclerotic organ damage [27]. The prevalence of MIA in CKD is associated with hypercatabolism, malabsorption due to overhydration and swelling of the gastrointestinal mucous, weakened appetite, loss of protein in the urine and during dialysis, as well as hormonal imbalances. Hormonal disturbances (especially those affecting insulin, insulin-like growth factor (IGF-1), adiponectin, ghrelin, and somatostatin (GH)) contribute to the state of chronic inflammation [26]. In ESRD patients, impaired balance between anti-inflammatory adiponectin and proinflammatory leptin is particularly visible [28]. The presence of proinflammatory cytokines is associated with disturbances of signal transduction and the development of insulin resistance, resulting in lipid metabolism disorders involving abnormal triglyceride metabolism, the rise in lipolysis with an enhanced release of free fatty acids (TNF-α, IL-6), and stimulation of ectopic lipid deposition (leptin) [29]. Moreover, insulin resistance is associated with endothelial dysfunction and an increase in blood pressure, which are both related to increased cardiovascular morbidity. Furthermore, the development of protein–energy wasting (PEW) is one of the strongest predictors of mortality in patients with CKD [26]. Systemic inflammation; uremic toxins; dialysis techniques promoting enhanced catabolism and systemic inflammation; and metabolic dysfunction involving the stimulation of appetite suppression hormones, such as insulin and leptin, which leads to diminished nutrient intake, are all of key importance for the development of PEW [30]. Among adverse effects of PEW there are: poor quality of life, sarcopenia, vascular calcifications, changes in lipid metabolism, increased inflammation and higher prevalence of cardiovascular disease, elevated rates of the number of hospitalizations, and increased mortality [31]. In ESRD, inflammation seems to be further aggravated by uremic immune dysfunction, deficient renal cytokine clearance, as well as inflammatory responses to dialysis. Some authors suggest that the improvement of ESRD patients’ prognosis is possible only as a result of the implementation of several actions targeted at tackling single causes of inflammation in the inflammatory cascade [32]. Apart from chronic inflammation in patients with CKD, especially with ESRD, as well as anemia, the rise in sympathetic tone, uremic toxin activity, aforementioned protein–energy malnutrition, endothelial dysfunction, calcium phosphate disorders, and pro-coagulation contribute to the accelerated development and progression of atherosclerosis [26]. Finally, ESRD is associated with modifications of lipid components, lipoproteins, and proteins. Dyslipidemia observed in ESRD is characterized by the hypertriglyceridemia, increase in IDL-C and chylomicron remnants, and a reduction in HDL-C and apolipoprotein (apo) AI (apoA-I) as well as apoC-II/apoC-III ratio [33,34]. Also, uremia-associated inflammation in ESRD can convert HDL from an antioxidant into a pro-oxidant particle [33]. Again, a vicious circle is observed in uremic patients and it involves reduced catabolism of intermediate-density lipoprotein (IDL) and LDL, which results in their longer residence time in plasma and further in alteration of the apolipoprotein B (apoB) contained in these lipoproteins via carbamylation, oxidation and glycation. Considering changed lipid subfraction turnover, the time of lipoproteins residence in the circulation of CKD patients is prolonged, which translates into higher risk of post-ribosomal modification of lipoproteins (such as glycation, oxidation, and carbamylation). According to studies, extensively modified lipoproteins show decreased affinity towards classic LDL-C receptors, thus they are captured by scavenger receptors (SR) on the surface of abundant macrophages, which in consequence promote atherosclerosis [34,35]. The mechanism underlying the oxidative stress in CKD patients is complex, multifactorial and not fully explained [26]. Due to the importance of oxidative stress in CKD patients, this review purpose is to shed some light on the mechanisms related to enhanced cardiovascular risk in patients with renal insufficiency. 

## 3. Oxidative Stress

The kidney is one of the vital sources of antioxidant enzymes, including glutathione peroxidases, and therefore, the impairment of this organ in the course of CKD is associated with decreased levels of these enzymes as well as increased levels of pro-oxidants [36]. The interplay between oxidants and antioxidants controls crucial pathways and cell metabolism [37,38]. In healthy conditions, reactive species are removed by natural endogenous defense mechanisms. However, in some diseases the defense mechanisms are impaired and/or the production of reactive species is so enhanced that it results in oxidative stress. Numerous studies confirmed the impairment of antioxidant systems (e.g., reduced activity of glutathione peroxidase and copper, zinc superoxide dismutase, and paraoxonase) in patients with CKD [39,40]. 

Oxidative stress is defined as the imbalance between the production of pro-oxidants and antioxidant defense mechanisms based on reactive oxygen species degradation. Reactive species generation and their cellular localization are usually in equilibrium with the availability of antioxidant enzymes including cystolic catalase (CAT), superoxide dismutase (SOD), and glutathione peroxidase (Gpx). The normal functioning of cells requires appropriate levels of both elements. In oxidative stress, the production of reactive oxygen species (ROS) exceeds the scavenging capacity of antioxidant systems [3,40]. The enhancement of oxidative stress has been demonstrated already in the early stages of CKD [41,42,43]. Numerous studies indicated that oxidative stress was significantly increased in patients with advanced renal impairment, but this state is exacerbated by hemodialysis [2,7,9,44]. Some authors suggested on the basis of in vivo studies that increased oxidative damage was the result of diminished levels of these enzymes rather than enhanced ROS production [45,46]. However, according to others, oxidative stress is associated with the production of highly reactive intermediates during inflammation; on the other hand, also reactive oxygen species (ROS) are able to stimulate pro-inflammatory mediators, such as NF-κB, thus promoting inflammatory response [41]. Inflammatory cells have been confirmed to be a source of free radicals, such as reactive oxygen and nitrogen species [47]. The upregulation of inflammatory markers observed in CKD patients (including platelet-derived growth factor and tumor necrosis factor-α) results in NADPH oxidase activation and subsequent generation of intracellular O_2_^•^ and H_2_O_2_ [48,49]. The presence of aggravated inflammatory state in CKD can stimulate the activation/recruitment of polymorphonuclear neutrophils and monocytes, which leads to stimulation of myeloperoxidase (MPO) and enhanced ROS production [50,51].

Uremic toxins have been shown to play a vital role in the onset of oxidative stress. Martinon et al. [52] demonstrated that uremic toxins promoted the development of inflammatory state and oxidative stress via priming acute inflammatory polymorphonuclear lymphocytes, stimulating interleukin (IL)-1β and IL-8. In turn, Sakamaki et al. [53] suggested that they stimulated the innate immune response through CD8+ cells. Stockler-Pinto et al. [54] revealed that indoxyl sulphate-related ROS production primarily resulted from the activation of nicotinamide adenine dinucleotide phosphate (NADPH) oxidase. Also, the synthesis of uric acid can aggravate oxidative stress via the activity of xanthine oxidoreductase, which generates reactive oxygen species [55]. However, some other reports suggest that in the presence of specific components, in various physiochemical circumstances and in different compartments of the human body, uric acid may play an anti-oxidant role in vivo [56]. Uric acid poses strong reducing and antioxidant properties, however, its elevated levels in CKD patients are believed to pose potential risk factors for CVD [57]. Under conditions of oxidative stress, high concentrations of uric acid have been shown to act as a pro-oxidant, particularly when antioxidant systems are impaired. However, it was also found to be cleared by HD as evidenced by a reduction in uric acid levels compared to pre-HD state [58]. 

The hemodialysis itself also enhances the oxidative stress due to the fact that antioxidant systems, particularly those of low or very-low molecular weight, are filtered during the procedure, and both the dialysis membrane and dialysate can activate leukocytes, leading to the aggravation of inflammation and enhanced ROS production [3]. Increased oxidative stress occurring in HD patients depends on many factors including aging, impairment of the residual renal function and subsequent uremic state, as well as the HD procedure itself [58]. During the initiation of the dialysis process, the membrane and dialysate induce inflammation and promote an important increase in ROS production. Post-dialysis, the levels of oxLDL have been shown to be elevated. However, post-dialysis, the activity of XOD and 8-OHdG levels are considerably diminished, which suggests that markers of oxidative stress are efficiently filtered during the dialysis process [3]. Also, markers of antioxidant defense decreased after HD [3]. Also, Liakopoulos et al. [59] stated that excessive oxidative stress in HD patients was related with the loss of antioxidants during the procedure and the accumulation of oxidative products. The level of oxidative stress was shown to be higher in ESRD patients on peritoneal dialysis (PD) compared to non-dialyzed uremic patients, however, it is lower in comparison to HD patients [59,60]. This observation was confirmed by Chen et al. [61] who demonstrated higher resting levels of superoxide anion in the whole blood after each HD session. Moreover, Granata et al. [62] revealed that patients with CKD and those undergoing hemodialysis show impaired mitochondrial respiration. The aggravation of oxidative stress can also be associated with the impaired activation of nuclear factor erythroid 2-related factor 2 (Nrf2), which is responsible for the regulation of genes encoding detoxifying and antioxidant proteins and enzymes (e.g., CAT, NAD(P)H dehydrogenase [quinone] 1 (NQO1), SOD [63]. Oxidative stress in patients with advanced stages of CKD can also be exacerbated by iron therapy, which is frequently used to treat anemia [36]. This phenomenon is associated with the fact that the administration of intravenous iron and the supersaturation of iron sequestration proteins (e.g., ferritin and transferrin) may result in the formation of free iron showing oxidative properties. 

Decreased NO production, the formation ofreactive nitrogen and oxygen species are responsible for the detrimental effects related to oxidative stress

### 3.1. Nitric Oxide (NO)

The kidney is an important source of L-arginine, which is a precursor for nitric oxide (NO); therefore, the decrease in its mass may result in diminished production of L-arginine and NO activity [47]. Due to the fact that nitric oxide is essential for vascular endothelial cell function, its decreased bioavailability is associated with endothelial dysfunction observed in hypertension, diabetes mellitus, atherosclerosis, and CKD [4]. Asymmetric dimethylarginine (ADMA), which is an endogenous amino acid resembling L-arginine, inhibits endothelial nitric oxide synthase, thus impairing NO synthesis. Elevated ADMA concentrations have been reported in ESRD. Moreover, Ravani et al. [64] suggested that elevated ADMA levels were a strong independent risk factor for the progression of CKD and patient mortality. O_2_^−^ and H_2_O_2_ are the precursors used for the production of even more powerful oxidants. The first of them shows the affinity towards free radical NO and their reaction results in the formation of peroxynitrite (ONOO^−^). The effects of both ONOO^−^ and hydroxyl (OH^−^) involve extensive nitrosative and oxidative modifications to proteins, lipids and nucleic acids [41].

### 3.2. Reactive Oxygen Species (ROS)

Reactive species produced in normal physiological state are inactivated by enzyme systems (e.g., glutathione) as well as other antioxidants (called scavengers) [41]. However, the excessive amount of ROS cannot be neutralized by scavenger systems, and therefore, they cause oxidative damage to proteins, nucleic acids and lipids; impair cellular activity; and hinder enzymatic activity [41]. In the kidneys, ROS are primarily synthesized by the mitochondrial respiratory chain and by enzymes such as NADPH oxidase (NOX) [41]. According to studies, NOX isoforms are vital players in the aggravation of oxidative stress, which results in the worsening of vascular function and promoting fibrosis [65,66]. To a lesser extent, ROS are produced by endoplasmic reticulum, peroxisomes and lysosomes [67]. Nox4 belonging to NADPH oxidase family is expressed in smooth muscle cells, vascular endothelial, as well renal proximal tubules, which explains why renal impairment may influence its expression or activity [68,69]. Also, pro-oxidant enzymes such as xanthine oxidase (XOD), in which activity has been shown to be considerably increased in uremia, is an additional possible source of ROS in CKD [3,70]. 

Reactive oxygen species (ROS) mainly include (O_2_^•−^), the hydroxyl radical (^•^OH) and hydrogen peroxide (H_2_O_2_) [47]. In healthy metabolic cells, their production is counteracted by mitochondrial or cystolic catalase (CAT) or thiol peroxidases, which catalyze H_2_O_2_ reduction into water and O_2_. Mitochondria comprise also other antioxidants, including manganese-SOD (Mn-SOD) and Gpx, which neutralize formed ROS. Mn-SOD converts O_2_^•−^ to H_2_O_2_, which in the next step is decomposed by CAT and Gpx [71]. In peroxisomes, the stabilization of O_2_^•−^ is related to the activity of copper/zinc-SOD (Cu/Zn-SOD) [72,73]. Also, glutathione homeostasis (Gpx, glutaredoxins, glutathione-S-transferase, peroxiredoxins and thioredoxins) is vital for maintaining cellular redox balance [47,74]. Xanthine oxidase (catalyzes the oxidation of hypoxanthine to uric acid, releasing in consequence ROS (O_2_^•^, ^•^OH, and H_2_O_2_) are by-products [51]. The formed uric acid accelerates CKD progression to renal failure and enhances the risk of cardiovascular events [75]. Reactive oxygen species are highly reactive and thus damage the variety of cellular structures and functional pathways [47]. Cellular H_2_O_2_ is rather stable, however, it still has potential to interact with numerous substances and cause destruction. Ferrous iron (Fe^2+^) can interact with H_2_O_2_, resulting in its cleavage and formation of the most reactive ^•^OH form [76]. Protein tyrosine phosphatases have been shown to be major targets for oxidant signaling due to the fact that they are greatly susceptible to oxidative modification of amino acid residue of cysteine [77]. 

The presence of oxidative stress and the impairment of antioxidative defense mechanisms in patients with CKD/ESRD have been confirmed in numerous studies. Some products of oxidative metabolism, including advanced glycation end products (such as pentosidine or advanced oxidation protein products), have been demonstrated to accumulate in renal failure [78,79]. Kinugasa E [80] demonstrated increased circulating levels of oxidative stress markers, including advanced glycation end products (AGEs), malondialdehyde (MDA) and 8-hydroxyde-oxyguanosine in blood and/or tissue in CKD patients. Advanced glycation end products (AGEs) acting via a specific receptor (RAGE) activate MAP kinase transduction pathway and in consequence lead to an increase in the level of pro-inflammatory cytokines, enzymes and adhesion molecules [81,82]. Colombo et al. [83] confirmed the existence of a relationship between uremia and oxidative stress, which was assessed on the basis of severe protein oxidative damage (including plasma advanced oxidation protein products) in end-stage renal disease (ESRD) patients on maintenance hemodialysis (HD). 

### 3.3. Consequences of Oxidative Stress 

Aggravated oxidative stress has been reported to be involved in the pathomechanisms of several diseases, including cardiovascular disease and chronic kidney disease. Elevated oxidative stress has been demonstrated to be strictly related to kidney and cardiac damage as it aggravates kidney dysfunction and induces cardiac hypertrophy, which is an independent risk factor for heart failure (HF) [43,51]. Oxidative stress has been shown to affect upstream transcriptional gene regulation. Numerous studies provided evidence that proliferator-activated receptors (PPARs), which play key roles in the transcriptional regulation of cell cycle progression, cell differentiation, glucose homeostasis, lipid metabolism, and inflammation, are altered in CKD and CVD [84,85,86]. Oxidative stress results in the damage of nucleic acids, including the modifications of bases (especially guanine in DNA) and covalent crosslinks, leading to single- and double-strand breaks. The oxidation of guanine is associated with the formation of oxidized products including 8-hydroxy-20-deoxyguanosine (8-OH-dG), which are highly prevalent in chronic and degenerative diseases, including CKD [87]. 

Oxidative stress is responsible for progressive renal damage, which in consequence may lead to renal ischemia, glomeruli damage, cell death and apoptosis, and further worsening of the severe inflammatory processes [41,88]. Fujii et al. correlated oxidative stress with glomerular abnormalities, including glomerular hypertrophy and mesangial proliferation, observed in the course of diabetic nephropathy [89]. It is also an infamous factor responsible for cardiac damage, such as hypertrophy, fibrosis, apoptosis, and remodeling [90]. Numerous mechanisms via which oxidation products promote vascular injury have been suggested [91,92,93,94]. NADPH oxidases, which are the major sources of ROS, participate in the pathogenesis of cardiac remodeling via its impact on redox-sensitive signal transduction [43]. Numerous studies confirmed that both the expression and the activity of NADPH oxidase were elevated in the myocardium of patients with ischemic and non-ischemic heart failure [95,96,97]. Moreover, higher activation of NADPH oxidase was involved in fibrosis and cardiac hypertrophy [98,99]. Uremic toxin, indoxyl sulphate, which promotes the production of ROS through the stimulation of NADPH oxidase or NADPH-like oxidase, has been shown to be involved in vascular disease, as it promotes vascular smooth muscle cell proliferation and vascular calcification; in addition, it is associated with higher mortality observed in CKD patients [100,101,102]. Moreover, this toxin also reduces levels of total glutathione in endothelial cells [43,103]. 

Oxidative stress-induced endothelial dysfunction and subsequent reduction in NO bioavailability promote the development of atherosclerosis. Peroxynitrite generated from NO is involved in numerous unfavorable vascular actions. The inactivation and the deficiency of NO resulting also from the actions of reactive species decrease the protection of kidney function, which is related to NO-dependent increase in renal blood flow, stimulation of pressure natriuresis, regulation of tubuloglomerular function, and maintenance of fluid and electrolyte homeostasis [41,104]. 

Oxidative stress is also associated with the formation of oxidized low density lipoprotein (ox- LDL), which play a crucial role in the pathogenesis of atherosclerosis [105]. The accumulation of oxidized low-density lipoproteins in arterial intima is the initial step of atherosclerotic process development [13]. Also, advanced glycation end products, in which production is enhanced in renal failure, exert atherogenic effects [13].

**Figure 1:**
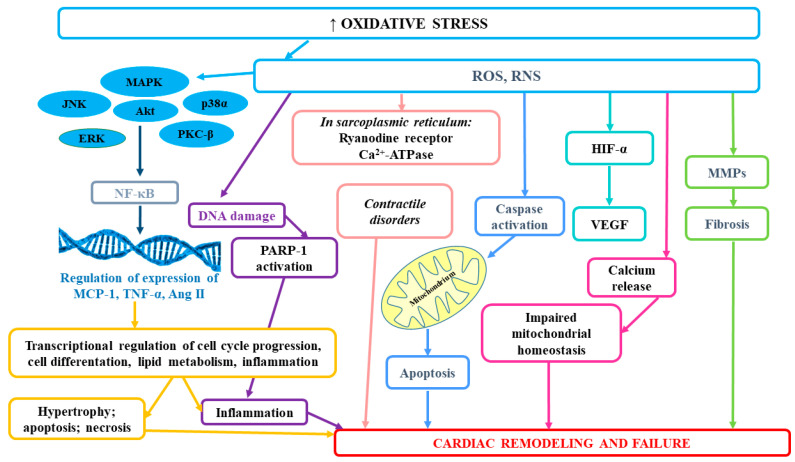
The role of oxidative stress in the development of cardiac remodeling and heart failure.

The link between cardiovascular disease and CKD may also involve the actions of the functional mitochondrial angiotensin system, which is regulated by oxidative stress [106]. Angiotensin type II receptors co-localized with angiotensin on the inner mitochondrial membrane of human mononuclear cells were shown to control mitochondrial NO production and respiration. The activation of the renin–angiotensin system (RAAS) in the course of renal impairment is involved in the process of left ventricular (LV) remodeling [107]. Ang II induces vasoconstriction and aldosterone release and it mediates hemodynamic alterations, which in consequence, lead to cardiac and vascular remodeling [108]. Furthermore, both angiotensin II and aldosterone actions involve the activation of mitogen-activated protein kinases (MAPKs), as well as c-Src and Ki-ras2A pathways engaged in the development of inflammation, in the production of O_2_ and H_2_O_2_, endothelial dysfunction, as well as hypertrophic growth [109,110]. Higher O_2_ levels are associated with enhanced protein kinase C (PKC) activity and NOS uncoupling, as well as consequent loss of vasodilation [51]. ONOO-associated loss of vasodilation and subsequent endothelial dysfunction play a vital role in the development of hypertension and further contributes to hypertrophic remodeling [111]. In CKD patients, oxidative stress leads also to left ventricular hypertrophy (LVH). The role of oxidative stress in the development of cardiac remodeling and heart failure has been summarized at Figure 1.

**Figure 2:**
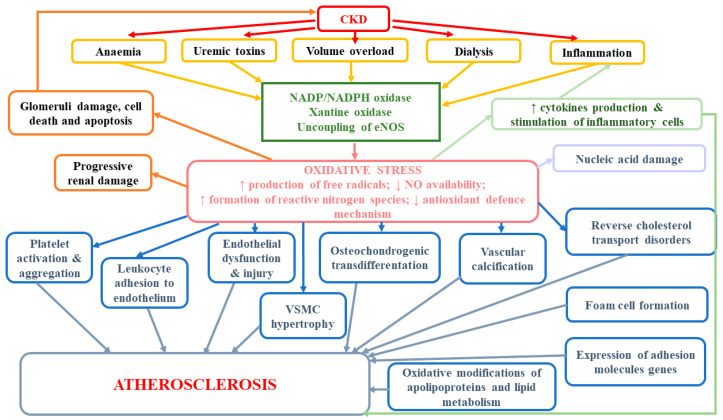
The role of oxidative stress in the development of atherosclerosis and other adverse consequences.

According to studies, oxidative stress as well as excessive ROS production are important factors mediating osteochondrogenic transdifferentiation of vascular smooth muscle cells (VSMCs) and enhanced vascular calcification [112]. The development of vascular calcifications (VC), which occurs commonly in CKD patients, exerts a direct impact on vessel functions and CVD development. Considerable leukocyte infiltration and the presence of IL-1β and MMP-1 have confirmed that human calcified areas in aortic valves lead to accelerated atherosclerosis, as well as higher rates of cardiovascular and all-cause mortality [113,114,115,116]. Oxidative stress has been demonstrated to contribute to the phenotype switch of vascular smooth muscle cells (VSMCs) even in early CKD [116]. Huang et al. [116] provided evidence for the existence of a kinetic relationship between oxidative stress and vascular calcification and osteoblastic transition. They observed that serum derived from patients with early stage CKD directly induced osteoblastic transition of primary rat VSMCs and calcium deposition in VSCMs, but it did not affect serum phosphorus level. In in vitro studies, hydrogen peroxide (H_2_O_2_) and xanthine/xanthine oxidase, which generates superoxide anion, were shown to boost osteochondrogenic transdifferentiation of VSMCs [112,117,118]. Intensified calcification in the presence of H_2_O_2_ was associated with higher expression of osteogenic markers, such as osteocalcin (OCN), runt-related transcription factor 2 (Runx2) and alkaline phosphatase (ALP), and lower expression of the contractile VSMCs phenotype markers, such as smooth muscle α-actin (α-SMA) and SM-22α [118]. It has been suggested that Msx2 is an important factor involved not only in transcriptional programming of osteoblastic lineage development but also in BMP-2-mediated vascular calcification through the activation of Wnt catenin signaling and β-catenin-induced activation of Pit1, a type III sodium-dependent phosphate cotransporter [119,120,121]. Cai et al. found that WNT/β-catenin signaling directly elicited osteogenic transdifferentiation and calcification of VSMCs though the modulation of Runx2 gene expression [122]. Huang et al. [116] suggested that the development of vascular calcification could be partly mediated by upregulation of NOX1 as well as ERK kinases as downstream events of NOX1-induced VC. Oxidative stress also indirectly may stimulate vascular calcification. It has been demonstrated that lipid oxidation products present in oxidized low-density lipoprotein rise the activity of ALP and promote calcification of vascular cells, which in consequence may lead to atherosclerosis-associated intimal calcification [123]. Moreover, it seems that oxidative stress in uremia enhances the formation of advanced oxidation protein products (AOPP), but at the same time, the accumulation of AOPP may pose a trigger for enhanced oxidative stress, which gives a positive feedback loop of elevated and maintained oxidative stress in uremic patients [124]. The results of in vitro study revealed that AOPP could directly stimulate osteoblast differentiation and calcification of smooth muscle cells [124]. You et al. [124] demonstrated that AOPP rose the calcium level in human aortic smooth muscle cells (HASMCs) (probably inducing their calcification) and considerably enhanced protein levels and mRNA expression of osteopontin (OPN), which may suggest that AOPP could promote osteoblast differentiation of HASMCs. Moreover, AOPP up-regulated mRNA expression of a transcription factor CBF-α1, which had earlier been found to increase the expression of osteoblast-specific genes, e.g., osteocalcin and alkaline phosphatase [125,126]. Finally, they were shown to considerably lower the expression of SM-α-actin expression [124]. According to in vitro studies, advanced oxidation products can not only trigger the oxidative burst of human monocyte and neutrophil but also induce enhanced production of oxidants by leukocytes [124,127,128]. It has been suggested that the mechanism of AOPP-stimulated smooth muscle cells differentiation may involve the activation of extracellular signal-regulated kinase (ERK), which is a part of MAPK pathway [124]. ERK is able to induce the osteoblast-related gene expression by extracellular matrix-integrin receptor interaction, bone morphogenetic protein 2 (BMP-2) and growth factors, thus leading to osteoblast differentiation [129,130,131]. Moreover, MAPK increases the expression of osteocalcin and AOPP-induced calcium deposition, which results also in the calcification of HASMC [130,132]. The role of oxidative stress in the development of atherosclerosis and other adverse consequences has been presented at Figure 2.

Finally, oxidative stress, especially the exposure to H_2_O_2_, has been demonstrated to alter membrane properties of red blood cells (RBC) and accelerate RBC removal in the spleen [51,133]. The increased susceptibility of RBCs to oxidative damage along with the higher risk of ROS production in iron deficiency anemia in CKD create a vicious cycle of enhanced RBC death, anemia and oxidative stress severity [51,133,134]. The lowering of hemoglobin content in iron deficiency-related anemia is associated with the decrease in partial pressure of oxygen, and this hypoxia-resembling state aggravates oxidative stress via auto-oxidation of hemoglobin to met-hemoglobin (metHb) with accompanying generation of O2 [135,136]. Iron deficiency also affects the expression of iron-containing endogenous antioxidant proteins e.g., peroxidase and catalase, as well as concentration of selenium, thus decreasing the activity of selenium-dependent enzyme GPx [137,138,139]. Therefore, it seems that timely intravenous iron replacement and the administration of antioxidants in clinical setting could improve CKD patients’ quality of life and decrease the risk of morbidity [140]. 

Numerous studies have confirmed that oxidative stress-related oxidation of fatty acid end-products (malondialdehyde) and serum albumin is associated with higher mortality in hemodialysis [141]. According to some authors, the decrease in antioxidant defense in hemodialysis results in enhanced all-cause and cardiovascular mortality in these patients [142,143,144]. Sangeetha Lakshmi et al. [58] revealed that the concentration of malondialdehyde (biomarker of oxidative stress) was considerably increased in patients with CKD and accompanying cardiovascular disease compared to patients with CKD but without cardiovascular disease. Substantial elevation of serum malondialdehyde levels observed in HD patients suffering from CVD, compared with those without CVD, indicated an association between oxidative stress and the development of atherosclerosis in these patients [9]. Juretic et al. [141] observed reduced PON in those patients with uremia who were at higher risk of cardiovascular disease, compared to persons with normal kidney function. It has been suggested that the loss of PON activity may increase the risk for oxidative stress and cardiovascular disease patients with chronic kidney disease, despite the lack of correlation with oxidized LDL [40]. Finally, Russa et al. [2] observed higher values of both oxidative stress and antioxidant barrier in hemodialysis patients with previous acute myocardial infarction compared to patients without cardiovascular events.

Enhanced risk of mortality risk might be mitigated by diminishing of oxidative stress, for example through the use of less aggressive types of dialysis (e.g., peritoneal) or antioxidant therapies [3]. Table 1 presents the results of selected articles concerning adverse impact of oxidative stress.

## 4. Treatment Aiming to Decrease the Risk Resulting from Oxidative Stress

According to studies, antioxidant therapies may prove to be beneficial since they can decrease oxidative stress, reduce uremic cardiovascular toxicity and improve survival [104]. Endogenous or dietary antioxidants have also been suggested to exert protective effects against inflammation and kidney damage in patients with CKD [41]. The application of mitochondrial-targeted antioxidant therapy resulted in the improvement of cardiac hypertrophy and diastolic dysfunction through the diminution of oxidative stress, which confirms the role of oxidative stress in the progression of heart failure [145]. Antioxidant supplementation with vitamins A, C, and E; β-carotene; or N-acetyl cysteine (NAC) seems to be beneficial in decreasing cardiovascular risk in hemodialysis patents [3,146]. Vitamin E is a powerful antioxidant exerting anti-inflammatory properties; it has been shown to interfere with cell membrane lipid peroxidation [147]. Observational clinical studies have shown that the intake of vitamin E (more than 100 IU/day), which inhibits oxLDL formation by hindering lipid peroxidation, reduced the rate of coronary events in hemodialysis [41,148,149]. Randomized placebo-controlled Secondary Prevention with Antioxidants of Cardiovascular Disease in End-stage Renal Disease (SPACE) trial revealed that in hemodialysis patients, the supplementation of alpha-tocopherol (800 IU) decreased cardiovascular disease endpoints and resulted in a substantial improvement of cardiovascular complications (myocardial infarction) [149]. Antioxidant therapy with DL-α-tocopherol has been revealed to improve left ventricular hypertrophy (LVH) and to decrease adverse changes within the myocardium in experimental CKD [150].

Vitamin C plays a significant antioxidative role as it can reduce ROS levels, thus providing protection against kidney oxidative damage and helping to maintain vascular and endothelial function [151]. Wang et al. [152] demonstrated that vitamin C (ascorbic acid) diminished oxidative damage, inflammation and renal injury in ischemia nephrotoxic acute kidney injury and rhabdomyolysis-induced renal injury. Deicher et al. observed deficiency of vitamin C (non-enzymatic antioxidant) in hemodialysis patients, which was associated with dietary restrictions and/or its loss during dialysis. Study of HD patients demonstrated that low plasma vitamin C levels predicted fatal and major non-fatal adverse cardiovascular events in this group [153]. Some studies indicate that patients with chronic kidney disease and ESRD patients should be administered a limited dose of daily vitamin C supplement of 75 mg for females and 90 mg for males [154]. However, Jankowska et al. [155] suggested that the supplementation of vitamin C might lead to oxalate accumulation and subsequent elevation in oxidative stress, and therefore, antioxidants administration may not always be the best alternative. 

In turn, vitamin D is vital not only for the homeostasis of calcium/phosphorus and skeletal health but also for renal functioning. The deficiency of this vitamin is frequently observed in CKD and ESRD and has been shown to contribute to the deterioration of renal function and increased morbidity and mortality in patients with CKD [56]. Some studies have demonstrated that the intake of vitamin D can reduce kidney injury by suppressing inflammation, fibrosis and apoptosis, via hindering multiple pathways crucial in kidney injury, including renin-angiotensin-aldosterone system (RAAS), NFκ-B, Wnt/β-catenin, and TGF- β/Smad signaling pathways [156,157,158]. 

Due to the fact that indoxyl sulphate stimulates oxidative stress and hastens the progression of CVD in CKD, the lowering of its concentration may prove beneficial in this group of patients. This suggestion was confirmed in several studies that demonstrated that the use of oral charcoal adsorbent, which decreases the levels of circulating uremic toxins, prevented histological and functional aggravation of CKD and suppressed oxidative stress and the advancement of cardiac damage in CKD [159,160,161]. In other studies, the decrease in heart and left ventricular volumes, cardiac fibrosis, as well as the attenuation of cardiac concentric change were observed in pre-dialysis CKD patients after AST-120 administration [162,163]. Taken together, these results suggest that the administration of AST-120 may become a useful option for improving cardiovascular health in CKD patients [43].

Also, melatonin (N-acetyl-5-methoxytryptamine) has been proven to be highly efficient in many disorders associated with oxidative stress and inflammation in experimental animals [164,165,166]. It is an endogenous neurohormone modulating sleep, immune function, circadian rhythm, and sexual behaviors, which exerts free radical scavenger, antioxidant and anti-inflammatory effects [165,167]. Due to the fact that it scavenges reactive oxygen and nitrogen species and enhances antioxidant defense systems, melatonin prevents tissue damage and hampers transcriptional factors of pro-inflammatory cytokines. Numerous studies have indicated that it indirectly decreases oxidative stress via stimulation of the expression and function of some antioxidant enzymes, enhancing the activities of antioxidative defense systems and glutathione as well as rising the efficacy of the mitochondrial electron transport chain [168,169,170]. CKD patients have been shown to have impaired night-time secretion of melatonin, which is further associated with higher stimulation of intrarenal renin–angiotensin system (RAS), leading to enhanced reactive oxygen species (ROS) production, sodium retention, inflammation, and fibrosis [171]. These pathologies accelerate the progression of CKD to end-stage renal disease (ESRD). The results of studies indicated that the supplementation with exogenous melatonin can reverse adverse changes, slow down the progression of kidney impairment, reduce blood pressure, and also help to maintain the bioavailability of nitric oxide by acting on melatonin receptor MT2 [172]. Studies on animal models indicated that prolonged administration of melatonin enhanced the expression of markers associated with decreased oxidative stress, inflammation and vasoprotection [173]. It has also been shown to improve cardiovascular function as well as renal, cardiac and cerebral damage [174].

The introduction of therapy based on the thiol-containing compound acetylcysteine has been demonstrated to diminish the toxic effects of ischemia reperfusion syndromes of the heart, kidney, liver, and lung and enabled the reduction in the risk of primary cardiovascular endpoint (fatal and non-fatal myocardial infarction) by 40% in hemodialysis patients [13]. Tepel et al. [13] revealed that after adjustment for age, baseline systolic and diastolic blood pressure, medications, smoking, and the duration of hemodialysis, in the study group treated with acetylcysteine, the survival related to the primary end point was higher compared with the control group. Moreover, they observed 30% decrease in cardiac events, 69% reduction in ischemic strokes, and 36% drop in peripheral vascular disease in the acetylcysteine group, however, the differences did not reach the level of statistical significance [13]. Due to the fact that acetylcysteine acts as a free-radical scavenger or as a reactive sulfhydryl compound, it enhances the reductive capacity of the cell ameliorating coronary and peripheral vascular function [175]. However, this antioxidant therapy proved ineffective in patients with heart failure without renal disease [176]. Therefore, it seems that the systemic oxidative stress in uraemic milieu plays a vital role in the development of cardiac disease in renal patients

Some studies indicated that in patients undergoing maintenance, HD plasma concentrations of CoQ10 are reduced, which suggests that CoQ10 supplementation could represent a great antioxidant therapy for these patients [177]. Randomized, double-blind, placebo-controlled study carried out by Rivara et al. [178] demonstrated that administration of CoQ10 (1200 mg daily) as an antioxidant therapy was safe and well tolerated in patients receiving MHD, and it resulted in a substantial, dose-dependent rise in plasma CoQ10 levels compared to placebo and considerably diminished plasma concentrations of F2-isoprostanes, which are considered a robust plasma marker of oxidative stress. 

Antioxidative properties have also been observed in the case of angiotensin-converting enzyme inhibitors and lipid-lowering agents [179,180,181].

## 5. Conclusions

Elevated cardiovascular morbidity and mortality in patients with end-stage renal failure remains to be a challenge in medicine. Numerous studies indicate that oxidative stress may play an important role in the development and progression of cardiovascular disease. However, antioxidant therapies seem to exert beneficial effects as they decrease cardiovascular risk and they bring hope for less cardiovascular complications in this group of patients.

## Figures and Tables

**Table 1:** The results of selected articles concerning the adverse impact of oxidative stress.

Type of Study	Study Group	Most Important Results	Ref
Prospective, randomized, placebo-controlled trial	134 HD patients randomly assigned either to receive acetylcysteine (600 mg BID) or placebo	Patients in the acetylcysteine group had 40% lower risk of reaching the primary end point (cardiac event, ischemic stroke, peripheral vascular disease) compared with the control group (relative risk, 0.60 [95% CI, 0.38 to 0.95], *p* = 0.03). Oxidized LDL was significantly lower in the acetylcysteine group compared with the control group (0.13 ± 0.22 arbitrary units vs. 0.55 ± 0.14 arbitrary units, *p* < 0.01). Conclusions: In hemodialysis patients, treatment with acetylcysteine reduces composite cardiovascular end points.	[13]
Case-control	244 nondiabetic patients with CKD (57 patients with stages 1 to 2 CKD and 187 patients with stages 3 to 5 CKD and 52 normotensive healthy subjects (controls)	LVH is already present in the early stages of renal disease. Strong relationship between elevated pulse pressure and LVH in those with more advanced CKD suggests that increased arterial stiffness might have a role for LVH well before the start of dialysis therapy.	[182]
Randomized placebo-controlled Secondary prevention with antioxidants of cardiovascular disease in end-stage renal disease (SPACE) trial	196 HD patients with pre-existing CAD randomized to receive 800 IU/day vitamin E or matching placebo	Fifteen (16%) patients assigned to vitamin E and 33 (33%) of those assigned to placebo had a primary endpoint (relative risk 0.46 [95% CI 0.27–0.78], *p* = 0.014); 5.1% patients assigned to vitamin E and 17.2% patients assigned to placebo had myocardial infarction (0.3 [0.11–0.78], *p* = 0.016). Conclusions: In hemodialysis patients with prevalent cardiovascular disease, supplementation with 800 IU/day vitamin E reduces composite cardiovascular disease endpoints and myocardial infarction.	[149]
A meta-analysis of prospective studies	30 articles reporting calcifications and cardiovascular end-points	The presence of calcifications increased the risk for any cardiovascular event. In a population with renal insufficiency, the event rate for all-cause mortality in patients with calcifications was more than five times higher than in patients without calcifications. The presence of calcifications had the highest predictive power for a cardiovascular or cerebrovascular event in subjects with renal insufficiency.	[114]
Randomized controlled trial	280 patients with CKD not on HD enrolled in the MASTERPLAN study	AAC occur commonly in populations of non-dialysis CKD patients. Calcification score ≥ 4 was associated with cardiovascular events; HR for cardiovascular events in the high calcification score group was 5.5 (95 % confidence interval 1.2–24.8), *p* = 0.03	[115]
Case-control and animal models	Patients from a clinical trial ChiCTR-OCH-14004447: (a) 24 HD patients vs. 13 healthy individuals (b) patients with CKD stage 2–3 (n = 30), patients with CKD5 (n = 30), and normal adults (n = 15)	A robust elevation in oxidative stress in HD patients vs healthy individuals; the elevation was higher in patients with VC than those without VC. Kinetic relationship among oxidative stress, osteoblastic transition and VC following CKD progression were indicated; the magnitude of osteoblastic transition did not further increase from E4wkCKD to E5wkCKD in rats, suggesting complex contributions of osteoblastic transition to OS-associated VC in early stage CKD. Serum OS levels were increased in both CKD2–3 and CKD5 patients compared to healthy controls, which is consistent with a role of OS in causing osteoblastic transition-mediated VC. This study supported a direct role of NOX1 in the induction of VC in patients with CKD. Conclusions: Oxidative stress plays a role in VC development in HD patients. OS without an increase in serum phosphorus concurrently exists with VC in patients with early stage CKD and in a rat model for early stage CKD. Serum from CDK2–3 patients with OS abnormalities and normal levels of serum phosphorus directly induce calcium deposition in primary VSMCs. Osteoblastic transition of VSMCs contributes to VC in CKD patients; the phenotype switch is in part enhanced by OS, NOX1, and ERK.	[116]
VSMC culture; Western Blot Analysis	N/A	H2O2 at concentrations of 0.1 to 0.4 mm induced osteogenic differentiation and calcification of VSMC in a concentration-dependent manner. Runx2, a key transcription factor for osteoblast and chondrocyte differentiation, was required for oxidative stress-induced VSMC calcification. Conclusions: Enhanced expression of Runx2 is sufficient to induce VSMC calcification. Activation of AKT signaling appears to mediate oxidative stress-induced Runx2 expression and activity during VSMC calcification.	[118]
Cell culture; animal model	N/A	Runx2 is a direct transcriptional target of WNT/β-catenin signaling pathway. WNT/β-catenin signaling could play a crucial role in promoting VSMCs osteogenic trans-differentiation and the development and progression of vascular calcification.	[122]
Human Smooth Muscle Cells (HASMC) Culture	N/A	AOPP increased the calcium content of HASMCs suggesting that AOPP can induce calcification of HASMCs AOPP considerably increased the protein and mRNA expression of OPN in HASMCs, indicating that AOPP can induce osteoblast differentiation of HASMCs; AOPP up-regulated the mRNA expression of CBF-α1 transcription factor. enhancing the expression of osteoblast-specific genes (osteocalcin, alkaline phosphatase). Conclusions: AOPP can trans-differentiate the HASMCs to osteoblast-like cells.	[124]

CRF—chronic renal failure; HD—maintenance hemodialysis; AAC—abdominal aortic calcification; HR—hazard ratio; AOPP—advanced oxidation protein products.
